# Traditional Chinese medicine-related interventions for allergic conjunctivitis and vernal keratoconjunctivitis: clinical evidence, mechanistic hypotheses, and ocular surface safety considerations

**DOI:** 10.3389/fmed.2026.1903422

**Published:** 2026-07-30

**Authors:** Ruoxi Liu, Sihan Li, Yang Zhao, Shumin Liu

**Affiliations:** 1 Heilongjiang University of Chinese Medicine, Harbin, Heilongjiang, China; 2 The Second Affiliated Hospital of Heilongjiang University of Chinese Medicine, Harbin, Heilongjiang, China; 3 Heilongjiang University of Finance and Economics, Harbin, Heilongjiang, China

**Keywords:** allergic conjunctivitis, immunomodulation, ocular surface safety, traditional Chinese medicine, vernal keratoconjunctivitis

## Abstract

Allergic conjunctivitis (AC) and vernal keratoconjunctivitis (VKC) are immune-mediated ocular surface diseases in which itching, conjunctival hyperemia, tearing, mucus secretion, epithelial injury and recurrence may substantially affect comfort and quality of life. Standard care is based on allergen avoidance, lubricants, topical antihistamines, mast-cell stabilizers, corticosteroids and topical immunomodulators when clinically indicated. Traditional Chinese Medicine (TCM) modalities are used in some clinical settings as adjunctive strategies, but their evidence base is heterogeneous and should not be interpreted as a substitute for established ophthalmic therapy. This structured narrative review synthesizes ocular clinical evidence, indirect mechanistic evidence and ocular surface safety considerations for TCM modalities in AC and VKC. Five bibliographic databases—PubMed/MEDLINE, Web of Science Core Collection, CNKI, Wanfang Data and VIP Database—were searched for literature published from 1 January 2010 to 31 May 2026. The database searches were supplemented by targeted Google Scholar searches, reference-list screening and citation tracking, while earlier landmark sources were retained when clinically or mechanistically relevant. The retrieved clinical evidence was concentrated in AC/SAC/PAC and VKC. No eligible AKC- or GPC-specific TCM clinical studies were identified within the search scope; these conditions are therefore discussed only for contextual and safety relevance. Oral herbal formulas and integrated TCM-standard therapy accounted for most of the clinical evidence, whereas herbal ophthalmic preparations, particularly Houttuynia eye drops, provided a smaller but more ocular-specific signal. Mechanistic claims involving IgE–mast-cell responses, Th2/eosinophilic inflammation, epithelial alarmins, NF-κB/NLRP3 signaling, oxidative stress and barrier dysfunction should be regarded as hypotheses requiring ocular-specific validation. For local herbal ophthalmic preparations, the safety threshold is high and includes sterility, endotoxin control, pH, osmolality, particulate matter, preservatives, botanical authentication, formulation stability and corneal epithelial safety. Adequately powered clinical studies using standardized diagnostic criteria, validated ophthalmic outcomes, longer follow-up periods, and prospective safety monitoring are needed to strengthen the evidence base.

## 1 Introduction

Ocular allergic diseases are common immune-mediated disorders of the conjunctiva and ocular surface. They include seasonal allergic conjunctivitis (SAC), perennial allergic conjunctivitis (PAC), vernal keratoconjunctivitis (VKC), atopic keratoconjunctivitis (AKC) and giant papillary conjunctivitis (GPC). These disorders overlap clinically but differ substantially in age distribution, chronicity, immune mechanisms, relationship to atopy or mechanical factors, and risk of corneal involvement. Mild-to-moderate SAC/PAC is usually dominated by IgE-mediated mast-cell activation and early-phase mediator release, whereas VKC and AKC more often include chronic type 2 inflammation, eosinophilic infiltration, epithelial remodeling and ocular surface barrier injury. GPC is commonly associated with contact lenses, ocular prostheses or other mechanical stimuli, with immune and mechanical components coexisting (1–21).

This revised review primarily focuses on AC/SAC/PAC and VKC, as these were the only ocular allergy subtypes for which direct clinical evidence of TCM-related interventions was identified. AKC and GPC are discussed only for contextual and safety considerations, and no efficacy conclusions are extrapolated to these conditions (Table 1).

**Table 1:** Disease scope and evidence status in the revised review.

Subtype	Typical manifestations	Dominant immune/clinical features	Status in this review
SAC/PAC	Itching, redness, tearing and chemosis; usually recurrent and mild-to-moderate	IgE-mast-cell activation, histamine release and early-phase responses	Primary clinical evidence domain for oral herbal formulas and integrated therapy
VKC	Severe itching, mucus, giant papillae and corneal risk; often pediatric or adolescent	Th2/eosinophilic inflammation, tissue remodeling and barrier injury	Primary ocular-specific evidence domain for Houttuynia eye-drop reports
AKC	Chronic allergic eye disease often associated with atopic dermatitis and corneal complications	Mixed IgE-mediated and cellular immune mechanisms	Contextual discussion only; no eligible direct TCM clinical studies identified
GPC	Giant papillae related to contact lens, prosthesis or foreign-body exposure	Mechanical stimulation plus immune reactions	Contextual discussion only; no eligible direct TCM clinical studies identified

Conventional treatment includes allergen avoidance, artificial tears, cold compresses, topical antihistamines, dual-action antihistamine/mast-cell stabilizers, nonsteroidal anti-inflammatory drugs, short-term corticosteroids and immunomodulators such as cyclosporine or tacrolimus when appropriate (1, 6, 7, 9, 13, 14, 17, 22–25). Although conventional management remains the cornerstone of evidence-based care, persistent symptoms, concerns about long-term safety and steroid-related adverse effects, together with cost, limited access to specialist care, and incomplete control of chronic inflammation have driven growing interest in adjunctive therapies, including TCM-related interventions.

The interventions considered here are not a single therapeutic entity. They include oral herbal formulas, proprietary Chinese medicines, integrated TCM-standard therapy, herbal ophthalmic preparations, fumigation, washing, atomization, warm compresses and acupuncture-related therapies. These modalities differ in route, formulation, regulatory risk, comparator relevance and safety requirements. They must therefore be evaluated separately rather than pooled under a single undifferentiated label (26–44).

## 2 Methods: structured search and narrative synthesis

This review was conducted as a structured narrative review with a descriptive, non-quantitative evidence map. It was not intended as a systematic review, meta-analysis, or formal certainty-of-evidence assessment. Because direct clinical evidence differed substantially among ocular allergy subtypes, the primary efficacy focus of this review was AC/SAC/PAC and VKC. AKC and GPC were not incorporated as direct efficacy evidence domains because no eligible TCM-specific clinical studies were identified within the search scope; they were retained only for contextual discussion and safety considerations. The search aimed to identify literature related to AC/SAC/PAC and VKC; TCM modalities; ocular surface inflammation; immunomodulatory mechanisms; ocular delivery; and safety. Literature published from 1 January 2010 to 31 May 2026 was considered, while earlier landmark ophthalmology, allergy or TCM sources were retained when clinically or mechanistically relevant. The final database search update was completed on 31 May 2026.

Databases included PubMed/MEDLINE, Web of Science Core Collection, CNKI, Wanfang Data and VIP Database. Google Scholar was used only as an additional source to identify potentially relevant records not captured by indexed database searches, rather than as a primary bibliographic database for reproducible indexing. Primary database searches combined ocular-allergy terms with TCM-related intervention terms. Additional literature relating to mechanisms, formulation, ocular surface safety, clinical guidelines, and contextual evidence was identified through supplementary Google Scholar searches, reference-list screening, and citation tracking during the narrative evidence synthesis. Table 2 summarizes the database-specific search strategies, database-specific search fields, search time span, and supplementary search procedures used in this review. Four targeted supplementary Google Scholar searches were conducted. The first 50 relevance-ranked results from each search were screened when available; when fewer than 50 results were returned, all available records were screened. In total, 135 records were screened.

**Table 2:** Database search strategy.

Database/source	Search strategy and source-specific use
PubMed/MEDLINE	#1 Disease/subtype terms: (“allergic conjunctivitis”[Title/Abstract] OR “ocular allergy”[Title/Abstract] OR “seasonal allergic conjunctivitis”[Title/Abstract] OR “perennial allergic conjunctivitis”[Title/Abstract] OR “vernal keratoconjunctivitis”[Title/Abstract] OR “atopic keratoconjunctivitis”[Title/Abstract] OR “giant papillary conjunctivitis”[Title/Abstract]) #2 TCM/intervention terms: (“Traditional Chinese Medicine”[Title/Abstract] OR “Chinese herbal medicine”[Title/Abstract] OR “Chinese medicine”[Title/Abstract] OR “herbal formula”[Title/Abstract] OR “herbal eye drops”[Title/Abstract] OR Houttuynia[Title/Abstract] OR Yupingfeng[Title/Abstract] OR Guominjian[Title/Abstract] OR acupuncture[Title/Abstract] OR fumigation[Title/Abstract] OR washing[Title/Abstract] OR atomization[Title/Abstract] OR “warm compress”[Title/Abstract]) Final set: #1 AND #2
Web of Science Core Collection	#1 Disease/subtype terms: TS = (“allergic conjunctivitis” OR “ocular allergy” OR “seasonal allergic conjunctivitis” OR “perennial allergic conjunctivitis” OR “vernal keratoconjunctivitis” OR “atopic keratoconjunctivitis” OR “giant papillary conjunctivitis”) #2 TCM/intervention terms: TS = (“Traditional Chinese Medicine” OR “Chinese herbal medicine” OR “Chinese medicine” OR “herbal formula*” OR “herbal eye drop*” OR Houttuynia OR Yupingfeng OR Guominjian OR acupuncture OR fumigation OR washing OR atomization OR “warm compress*”) Final set: #1 AND #2
CNKI	#1 Disease/subtype block: SU = (‘过敏性结膜炎’ + ‘变态反应性结膜炎’ + ‘春季角结膜炎’ + ‘春季卡他性结膜炎’ + ‘特应性角结膜炎’ + ‘巨乳头性结膜炎’) #2 TCM/intervention block: (‘中医’ + ‘中药’ + ‘方剂’ + ‘鱼腥草’ + ‘玉屏风’ + ‘过敏煎’ + ‘针刺’ + ‘熏洗’ + ‘雾化’ + ‘冷敷’) Final executed expression: SU = (‘过敏性结膜炎’ + ‘变态反应性结膜炎’ + ‘春季角结膜炎’ + ‘春季卡他性结膜炎’ + ‘特应性角结膜炎’ + ‘巨乳头性结膜炎’) * (‘中医’ + ‘中药’ + ‘方剂’ + ‘鱼腥草’ + ‘玉屏风’ + ‘过敏煎’ + ‘针刺’ + ‘熏洗’ + ‘雾化’ + ‘冷敷’)
Wanfang Data	#1 Disease/subtype block: (过敏性结膜炎 OR 变态反应性结膜炎 OR 春季角结膜炎 OR 春季卡他性结膜炎 OR 特应性角结膜炎 OR 巨乳头性结膜炎) #2 TCM/intervention block: (中医 OR 中药 OR 方剂 OR 鱼腥草 OR 玉屏风 OR 过敏煎 OR 针刺 OR 熏洗 OR 雾化 OR 冷敷) Final executed expression: (过敏性结膜炎 OR 变态反应性结膜炎 OR 春季角结膜炎 OR 春季卡他性结膜炎 OR 特应性角结膜炎 OR 巨乳头性结膜炎) AND (中医 OR 中药 OR 方剂 OR 鱼腥草 OR 玉屏风 OR 过敏煎 OR 针刺 OR 熏洗 OR 雾化 OR 冷敷)
VIP Database	#1 Disease/subtype block: (M = (“过敏性结膜炎” OR “变态反应性结膜炎” OR “春季角结膜炎” OR “春季卡他性结膜炎” OR “特应性角结膜炎” OR “巨乳头性结膜炎”) OR R = (“过敏性结膜炎” OR “变态反应性结膜炎” OR “春季角结膜炎” OR “特应性角结膜炎” OR “春季卡他性结膜炎” OR “巨乳头性结膜炎”)) #2 TCM/intervention block: (M = (“中医” OR “中药” OR “方剂” OR “鱼腥草” OR “玉屏风” OR “过敏煎” OR “针刺” OR “熏洗” OR “雾化” OR “冷敷”) OR R = (“中医” OR “中药” OR “方剂” OR “鱼腥草” OR “玉屏风” OR “过敏煎” OR “针刺” OR “熏洗” OR “雾化” OR “冷敷”)) Final executed expression: #1 AND #2
Google Scholar, reference-list screening, and citation tracking	Used as a supplementary source rather than as a primary indexed bibliographic database. Four targeted supplementary searches were conducted to identify mechanistic, pharmacological, formulation, ocular surface safety, guideline, and contextual literature not captured by the primary database searches. Reference-list screening and citation tracking were additionally used during the narrative evidence synthesis. The first 50 relevance-ranked results for each supplementary query were screened when available
Time span (index date)	1 January 2010–31 May 2026; final search update: 31 May 2026

Numbered search blocks (#1 and #2) are presentation labels used to show how each database-specific expression was constructed; the complete expression represented by these blocks was executed.

Eligible sources included clinical studies, meta-analyses, systematic reviews, protocols, narrative reviews, pharmacological studies, mechanistic experimental studies, clinical guidelines and safety/formulation-related reports. Non-peer-reviewed popular-science webpages, patent materials and commercial product pages were not considered efficacy evidence. When relevant, they were considered only as background information for formulation translation and clinical-use context.

Ocular clinical evidence was defined as evidence derived from clinical studies, meta-analyses or clinical reports conducted in patients with AC, SAC/PAC or VKC. Indirect mechanistic evidence was defined as evidence from non-ocular allergic disease models, general inflammation models, pharmacological studies, network pharmacology or *in vitro* systems (44–46). Such evidence was used solely to support biological plausibility and was not interpreted as proof of clinical efficacy in ocular allergy.

**Figure 1:**
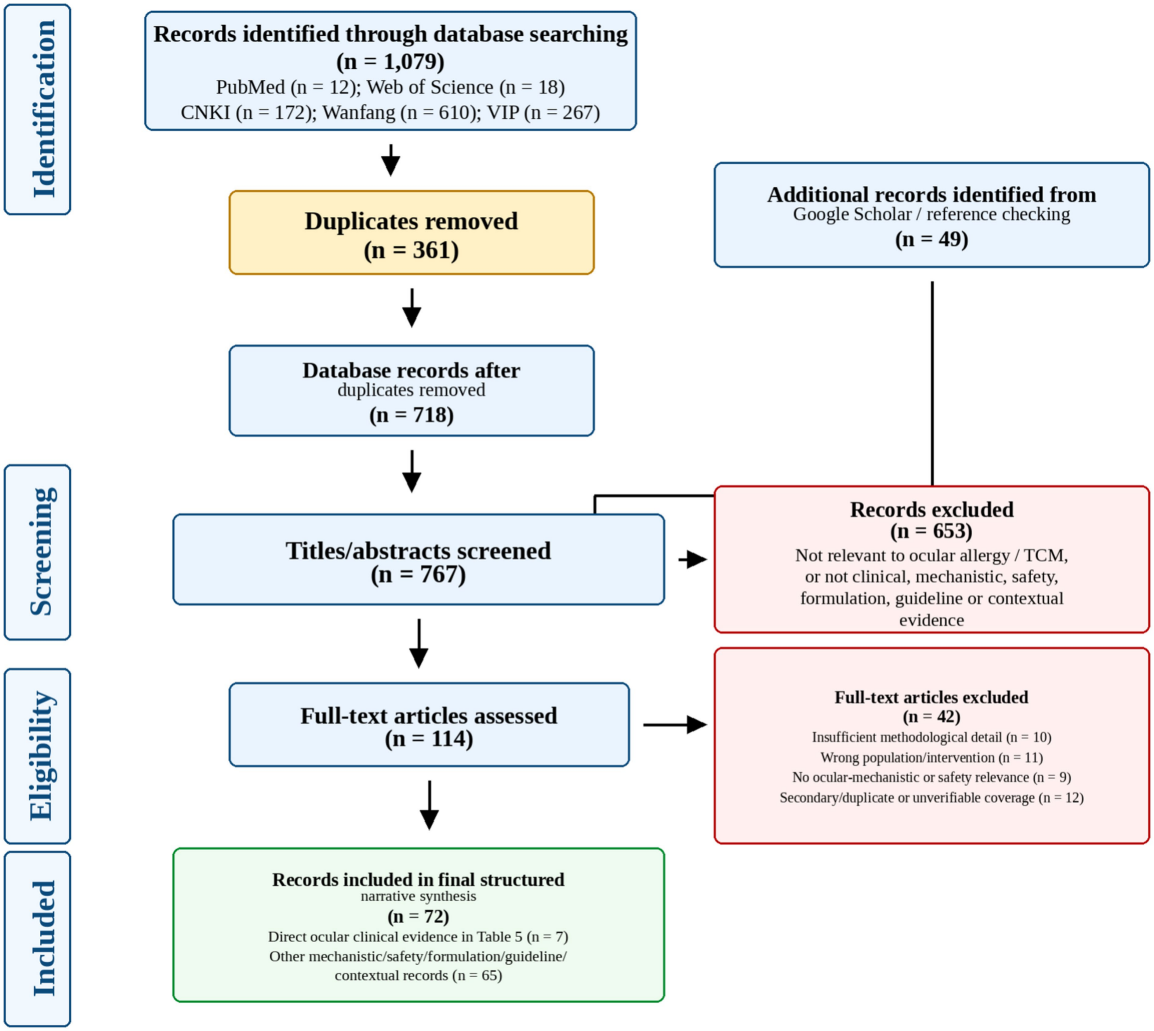
Study-selection flow diagram for the structured narrative review.

Two authors independently screened records and extracted information on disease subtype, intervention modality, intervention composition, dose or regimen, treatment duration, comparator, outcome measures, adverse-event reporting, unit of analysis and study limitations. Disagreements were resolved through discussion. The search identified 1,079 records across five bibliographic databases: PubMed/MEDLINE (*n* = 12), Web of Science Core Collection (*n* = 18), CNKI (*n* = 172), Wanfang Data (*n* = 610) and VIP Database (*n* = 267). After merging the database records, 361 duplicates were removed, leaving 718 unique records. Supplementary Google Scholar searches and reference-list screening identified 49 additional non-duplicate records after assessment of 135 relevance-ranked results, resulting in 767 records for title and abstract screening. Of these, 653 records were excluded, and 114 articles underwent full-text assessment. Forty-two full-text articles were subsequently excluded, leaving 72 records for the final structured narrative synthesis. Of these, seven core ocular clinical evidence records are summarized in Table 3. The study-selection workflow is shown in Figure 1. Because of substantial heterogeneity in disease subtype, intervention, outcome definition, study design, blinding, follow-up, safety reporting and unit of analysis, no *de novo* meta-analysis or formal certainty-of-evidence grading was performed.

**Table 3:** Clinical evidence summary table: Core clinical evidence for TCM modalities in allergic conjunctivitis and vernal keratoconjunctivitis.

Study	Core clinical evidence fields
Fang et al. 2022 (34)	Design: Meta-analysis record; detailed methods partly verifiable from accessible abstract and journal record Journal record: China Modern Doctor 2022;60(16):136–139 + 157. Sample size: 7 studies involving 610 participants according to the accessible CNKI abstract Unit of analysis: Dependent on included primary trials; patient-level versus eye-level handling not verifiable from accessible record Disease subtype: AC; SAC/PAC distinction not reported Diagnostic criteria: Varied across Chinese trials Intervention/dose or regimen: Modified Yupingfeng + Western pharmacotherapy; regimens varied Comparator: Western pharmacotherapy alone Duration: Varied across included studies Methodological details: Chinese-language meta-analysis using comparative clinical studies of modified Yupingfeng plus Western pharmacotherapy versus Western pharmacotherapy alone; the accessible abstract reports pooled total effective rate and recurrence, but does not provide a complete risk-of-bias table, model-selection rationale, heterogeneity values, allocation details of the primary trials or the full included-study list. Outcome measures: The accessible abstract reported pooled findings favoring modified Yupingfeng plus Western pharmacotherapy for total effective rate and recurrence. Because key methodological details remain incompletely transparent and potential overlap with primary studies in this table cannot be excluded, these findings were interpreted only as supportive secondary evidence rather than independently verifiable evidence. Adverse-event reporting: One mild diarrhea event was mentioned in the accessible record; adverse-event ascertainment appeared inconsistent or incompletely described Key limitations: Heterogeneous formulas/controls/diagnoses; non-standard total effective rate; original trial quality, unit of analysis and ocular-safety monitoring incompletely transparent; potential overlap with primary studies summarized below could not be excluded
Chen 2013 (48)	Design: Reported as randomized clinical study; PubMed-indexed; random-sequence generation, allocation concealment and blinding were not fully verifiable from the accessible report Sample size: 118 patients; treatment 74, control 44 Unit of analysis: Patients Disease subtype: AC with recurrent/perennial features Diagnostic criteria: Allergic conjunctivitis; recurrent/perennial symptoms and exclusion criteria reported in accessible full text Intervention/dose or regimen: Sodium cromoglicate eye drops + oral Yupingfeng granules; oral granules three times daily for 7 consecutive days in the report Comparator: Sodium cromoglicate eye drops alone Duration: Assessment at 2 weeks; oral granules administered for 7 consecutive days Outcome measures: Symptoms/signs improved in both groups; total efficacy 91.9% vs. 75.0% with sodium cromoglicate alone (*p* < 0.05) Adverse-event reporting: No significant adverse reactions reported Key limitations: Short follow-up; total efficacy is non-standard; allocation concealment/blinding and prospective ocular-safety protocol not fully transparent; corneal/formulation safety not assessed
Wang et al. 2022 (35)	Design: Reported as randomized clinical study; random-sequence generation, allocation concealment and blinding not confirmed in accessible abstract Sample size: 160 patients/320 eyes; 80 patients/160 eyes per group Unit of analysis: Patients and eyes; both-eye inclusion reported; adjustment for inter-eye correlation not described in accessible abstract Disease subtype: AC Diagnostic criteria: Hospital-diagnosed AC Intervention/dose or regimen: Tiaoti Tuomin Decoction orally + ocular cold-wet compress; individualized modifications require full-text confirmation Comparator: 0.05% azelastine hydrochloride eye drops Duration: 14 days Outcome measures: Symptom/sign scores, quality of life and serum IgG/IgA/IgE; total effective rate 92.5%; serum IgE decreased Adverse-event reporting: Detailed adverse-event monitoring not described in accessible abstract Key limitations: Single-center short-course study; combined oral formula + compress limits component attribution; serum markers not eye-specific; eye-level independence uncertain
Yu et al. 2021 (36)	Design: Reported as randomized pediatric clinical observation; random-sequence generation, allocation concealment and blinding not confirmed in accessible metadata Sample size: 60 children; 30/60 eyes per group as reported Unit of analysis: Children and eyes; eye-level independence and adjustment for inter-eye correlation not verifiable Disease subtype: Pediatric AC Diagnostic criteria: Outpatient pediatric AC Intervention/dose or regimen: Zhaqu Pingwei San + Guominjian with modifications; exact dose schedule not in public abstract Comparator: Azelastine hydrochloride eye drops Duration: 7 days/course; course number/follow-up require full-text confirmation Outcome measures: Clinical response; total effective rate reportedly higher in treatment group Adverse-event reporting: Adverse-event monitoring not described in accessible metadata Key limitations: Small pediatric single-center report; dose/course/follow-up and prospective adverse-event monitoring require full-text confirmation; limited validated ocular endpoints; eye-level independence uncertain
Xu and Cai 2019 (27)	Design: Comparative clinical study; allocation method and blinding unclear Sample size: 926 VKC patients: Houttuynia 276; olopatadine 305; combination 345 Unit of analysis: Patients; handling of bilateral eye data not clearly reported if both eyes were included Disease subtype: VKC Diagnostic criteria: Clinical VKC diagnosis Intervention/dose or regimen: Houttuynia eye drops 1 drop six times daily; olopatadine 1 drop twice daily; or combined regimen Comparator: Houttuynia alone and olopatadine alone groups Duration: 14 days; baseline, 1 h, 7 d, 14 d Outcome measures: Combination showed higher excellent/effective rates and faster symptom relief at several time points Adverse-event reporting: No obvious adverse reactions reported; formulation-level safety testing not fully described Key limitations: Short follow-up; unclear allocation/blinding; non-standard response rates; formulation and corneal epithelial safety parameters not fully characterized; bilateral-eye handling not transparent
Hang 2013 (38)	Design: Reported as randomized clinical study; random-sequence generation, allocation concealment and blinding not fully verifiable from accessible report Sample size: 63 patients/126 eyes; 32 patients in the combination group and 31 patients in the control group Unit of analysis: Patients and eyes; both-eye inclusion reported; adjustment for inter-eye correlation not evident Disease subtype: AC Diagnostic criteria: Symptoms/signs of AC; recent antihistamine/corticosteroid use and other ocular disease excluded Intervention/dose or regimen: Pemirolast potassium twice daily + Houttuynia four times daily; interval >10 min Comparator: Pemirolast potassium twice daily alone Duration: 14 days Outcome measures: Symptoms/signs improved more with combination; symptom score 1.22 ± 0.61 vs. 4.25 ± 0.84; sign score 1.21 ± 0.60 vs. 5.78 ± 1.29; effective rate 78.12% vs. 32.25% Adverse-event reporting: No structured adverse-event table; detailed prospective ocular-safety protocol not evident Key limitations: Small single-center study; short duration; non-standard total effective rate; limited ocular surface safety monitoring; eye-level independence uncertain
Huang et al. 2015 (47)	Design: Reported randomized eye-based comparative study; random-number-table allocation was reported, but allocation concealment, blinding, the unit of randomization and handling of inter-eye correlation were not described Sample size: 160 eyes; combination 80 eyes; control 80 eyes Unit of analysis: Eyes; the number of unique patients, bilateral-eye inclusion and adjustment for inter-eye correlation were not reported Disease subtype: AC Diagnostic criteria: Allergic conjunctivitis, including perennial, seasonal and contact allergic conjunctivitis; prespecified inclusion and exclusion criteria were reported Intervention/dose or regimen: Olopatadine eye drops, 1 drop twice daily, plus *Houttuynia cordata* eye drops, 1 drop twice daily; interval >10 min Comparator: Olopatadine eye drops, 1 drop twice daily Duration: 14 days Outcome measures: Symptom and sign scores decreased more with combination therapy; total effective rate 77.50% vs. 37.50% Adverse-event reporting: No obvious ocular worsening or systemic allergic reactions were reported Key limitations: Single-center, 14-day, eye-based study; allocation concealment and blinding not reported; non-standard total effective rate; formulation and corneal safety testing not detailed; number of unique patients, bilateral-eye inclusion and inter-eye correlation handling not transparent

Study-design labels were assigned conservatively. For studies reporting both patients and eyes, findings should be interpreted descriptively unless adjustment for inter-eye correlation was explicitly reported.

This figure summarizes the literature identification, screening, eligibility assessment, and inclusion process for the present structured narrative review. It is provided to improve methodological transparency and reproducibility and should not be interpreted as a PRISMA flow diagram for a prospectively registered systematic review or quantitative meta-analysis.

## 3 Evidence landscape across disease subtypes and TCM modalities

The available evidence was unevenly distributed across ocular allergy subtypes and TCM intervention modalities. Direct ocular clinical evidence was mainly concentrated in AC/SAC/PAC and VKC. VKC formed a smaller but more directly ocular clinical evidence stream, largely because of published clinical reports on Houttuynia eye drops. No eligible AKC- or GPC-specific TCM clinical studies were identified within the search scope. This absence should not be read as proof that such studies do not exist anywhere, but it does mean that this review cannot support efficacy claims for AKC or GPC.

To avoid implying quantitative grading, the distribution of direct clinical evidence is summarized as a non-quantitative matrix in Table 4 rather than as a figure. The matrix is limited to AC/SAC/PAC and VKC, because no eligible AKC- or GPC-specific TCM clinical studies were identified within the search scope; AKC and GPC are discussed in the text only as related disease contexts and safety-relevant comparators.

**Table 4:** Non-quantitative evidence matrix for TCM modalities in allergic conjunctivitis and vernal keratoconjunctivitis.

Modality	AC/SAC/PAC	VKC	Interpretation
Oral herbal formulas	Direct clinical reports; main body of oral-formula evidence	No eligible direct clinical reports identified	Evidence primarily relates to AC/SAC/PAC, with no eligible direct oral-formula studies identified in VKC
Integrated TCM-standard therapy	Direct clinical reports, often combined with standard topical antiallergic therapy	Limited reports	Pragmatic clinical approach, but treatment effects cannot be readily attributed because herbal and conventional therapies are combined
Herbal ophthalmic preparations	Limited direct reports	Direct VKC reports, mainly Houttuynia eye drops	Most directly local ocular domain, but trial quality and formulation safety remain major limitations
Fumigation/washing/atomization/warm compress	Exploratory reports	Exploratory or indirect reports	Require further standardization of composition, temperature, exposure, hygiene and ocular surface safety
Acupuncture-related approaches	Indirect evidence from non-ocular allergic diseases (eg, allergic rhinitis and asthma)	No robust direct ocular evidence	Discussed only as indirect systemic or neuroimmune plausibility, not as established ocular evidence

The matrix summarizes where direct or indirect evidence was identified within the structured narrative search. It does not represent study counts, therapeutic ranking, disease prevalence or certainty-of-evidence grading. It is intended only to illustrate the distribution of evidence across disease subtypes and intervention modalities.

Across the retrieved clinical literature, oral Chinese herbal medicine and integrated TCM-standard therapy constituted the largest body of direct clinical evidence. Representative AC-related clinical records included Yupingfeng-related prescriptions, Guominjian-related formulas, Tiaoti Tuomin formula, Zhaqu Pingwei San combined with Guominjian, integrated TCM-standard therapy and Houttuynia-containing topical regimens (34–38, 47, 48). Herbal ophthalmic preparations formed a smaller but clinically important topical domain, with Houttuynia eye drops being the most frequently reported example (26, 27, 38, 47). External ocular surface strategies such as fumigation, washing, atomization and warm compresses were less well represented and should be regarded as exploratory rather than established clinical interventions. Table 5 classifies these modalities by evidence position, comparator, commonly reported outcomes and safety concerns.

**Table 5:** Modality-specific classification of interventions considered in this review.

Modality	Evidence position	Typical comparator	Main outcomes reported	Main safety concerns
Oral herbal formulas	Principal body of direct clinical evidence, primarily for AC/SAC/PAC	Standard topical therapy, conventional care or before-after comparison	Total effective rate, symptom score, recurrence	Botanical identity, dose standardization, hepatic/renal toxicity, pregnancy risk, herb-drug interactions
Integrated TCM-standard therapy	Common clinical approach; oral or external TCM used as an adjunct to standard topical antiallergic therapy	Standard topical therapy alone	Total effective rate, symptom/sign scores, recurrence	Attribution of effect is difficult; combined safety monitoring is required
Herbal ophthalmic preparations	Smaller but more ocular-specific body of direct clinical evidence; Houttuynia eye drops are representative	Olopatadine, pemirolast potassium or combined topical therapy	Itching, hyperemia, symptom/sign scores, excellent/effective rates	Sterility, endotoxin, pH, osmolality, particles, preservatives, corneal epithelial toxicity, formulation stability
Fumigation/washing/atomization/warm compress	Exploratory and under-standardized	Local conventional care or adjunctive use	Comfort, itching, redness, local tolerability	Temperature, exposure time, contamination, particles, pH/osmolality and corneal safety
Acupuncture-related approaches	No robust direct ocular clinical evidence; most available evidence is indirect from non-ocular allergic diseases (eg, allergic rhinitis and asthma)	Variable	Indirect allergic outcomes rather than validated ocular endpoints	Standardization, adverse-event reporting, and avoidance of over-extrapolation to ocular disease

## 4 Core clinical evidence summary table

The review-level evidence map is insufficient on its own to support clinical interpretation. Table 3 provides a structured summary of the core ocular clinical evidence records identified in this review. It summarizes the direct ocular clinical evidence record by record and contains study design, sample size, ocular allergy subtype, diagnostic criteria, intervention composition and dose or regimen, duration, comparator, outcome measures, adverse-event reporting and key limitations. Records for which essential methodological details could not be extracted from the accessible report are not treated as core clinical evidence in this table; they are mentioned descriptively only as supporting clinical reports. When a specific item was unavailable despite targeted checking, this is stated explicitly rather than inferred. For several older Chinese clinical reports, complete methodological details were either unavailable from accessible records or not reported explicitly in the original publication; such records were not used to support strong efficacy claims.

This table is intended to improve verifiability, not to imply equal evidence quality. Study-design labels were assigned conservatively. Studies were not classified as confirmed randomized trials unless random-sequence generation and other core design features were verifiable from the accessible report. When randomization was stated but random-sequence generation, allocation concealment or blinding could not be confirmed, the record was described as “reported as randomized” rather than as a confirmed randomized trial. Meta-analyses, reported randomized studies, comparative clinical studies and uncontrolled clinical records have different evidentiary implications, and total effective rate is treated as a non-standard outcome requiring cautious interpretation.

Each study was summarized in a standardized format. Missing methodological details were stated explicitly rather than inferred. For studies reporting both patients and eyes, findings were interpreted descriptively unless adjustment for inter-eye correlation was explicitly reported. Some primary studies summarized in Table 3 may have been included in the Fang et al. meta-analysis; therefore, the secondary meta-analysis and the individual primary studies should not be interpreted as completely independent sources of evidence.

## 5 Modality-specific synthesis of clinical evidence

### 5.1 Oral herbal formulas

Oral Chinese herbal medicine represented the largest body of clinical evidence identified in the retrieved literature. It was used either alone or as the systemic component of integrated TCM-standard therapy (34–37, 48). Fang et al. (34) reported a Chinese-language meta-analysis comparing modified Yupingfeng combined with Western pharmacotherapy versus Western pharmacotherapy alone for allergic conjunctivitis. According to the accessible CNKI abstract, the meta-analysis included seven studies involving 610 participants. The abstract reported pooled estimates favoring modified Yupingfeng plus Western pharmacotherapy for total effective rate and recurrence, with one mild diarrhea event reported. However, important methodological details, including the complete risk-of-bias assessment, model-selection rationale, heterogeneity statistics, allocation procedures of the included primary studies and the full study list, were not available from the accessible record. Therefore, this meta-analysis was regarded as supportive secondary evidence rather than definitive evidence, and potential overlap with the primary studies summarized in Table 3 could not be excluded. Within TCM theory, herbal formulas are commonly interpreted in terms of wind-induced itching, defensive-qi deficiency, damp-heat, recurrence tendency and allergic constitution. Although these concepts are clinically meaningful within the TCM framework, they do not constitute biomedical mechanisms.

Modern biomedical interpretations have focused on IgE production, mast-cell activation, Th1/Th2 immune balance, eosinophilic inflammation and cytokine regulation. However, ocular-specific biomarkers were rarely evaluated in the available clinical studies. Consequently, evidence supporting oral herbal formulas should be regarded as clinically suggestive rather than mechanistically confirmed in ocular allergy.

### 5.2 Integrated TCM-standard therapy

Integrated TCM-standard therapy, typically combining oral herbal medicine with standard topical antiallergic eye drops, represented the most common clinical management pattern identified in the retrieved literature. This pragmatic approach reflects routine clinical practice but makes it difficult to determine whether any additional benefit is attributable to the herbal intervention itself, improved adherence, outcome definitions, or other co-interventions.

Integrated therapy should be framed as a pragmatic adjunctive strategy rather than evidence that a specific herbal formula has independent antiallergic ocular efficacy. Future trials should include stable background standard therapy, prespecified rescue-medication rules, recurrence after discontinuation and validated ocular endpoints. A PubMed-indexed study reported as randomized evaluated sodium cromoglicate eye drops combined with oral Yupingfeng granules in 118 patients and reported a higher total efficacy rate than sodium cromoglicate alone, with no significant adverse reactions. However, this study was also limited by the use of non-standard response outcomes, short follow-up, and incomplete reporting of key methodological safeguards, including random-sequence generation, allocation concealment and blinding (48).

### 5.3 Herbal ophthalmic preparations and Houttuynia eye drops

Direct clinical reports are available in VKC and AC (27, 38, 47), and a related systematic-review protocol has also been published (26). The available evidence is best regarded as preliminary clinical evidence rather than confirmatory evidence.

The largest identified VKC study included 926 patients treated at the Eye Hospital of Wenzhou Medical University between July 2011 and January 2017. Patients received Houttuynia eye drops, olopatadine hydrochloride eye drops, or the combination regimen; treatment lasted 14 days and outcomes were compared at baseline, 1 h, 7 days and 14 days after administration (27). The combined regimen was reported to provide faster and greater symptom relief than either agent alone. The study was short, randomization and blinding were not clearly reported, and the outcome framework relied on excellent/effective rates rather than internationally standardized ocular endpoints.

A smaller study reported as randomized in AC compared pemirolast potassium eye drops alone with pemirolast potassium plus *Houttuynia cordata* eye drops. Sixty-three patients were allocated into a 31-patient control group and a 32-patient treatment group. The intervention group received pemirolast twice daily plus Houttuynia eye drops four times daily for 14 days, while the control group received pemirolast alone. Symptom and sign scores improved more in the combination group and the reported total effective rate was 78.12% compared with 32.25% in the control group (38). Despite reported randomization, the small sample size, short follow-up, use of total effective rate, unclear handling of two-eye data and limited safety information prevent strong conclusions.

These studies justify continued investigation of Houttuynia eye drops, but they do not establish a class effect for herbal ophthalmic preparations. Any topical herbal product should be evaluated as an ophthalmic formulation, not as a simple herbal extract. Necessary translational requirements include botanical identification, marker-compound characterization, sterility, endotoxin control, pH, osmolality, particulate matter, preservative toxicity, formulation stability, container-closure integrity and corneal epithelial safety.

### 5.4 External ocular surface strategies

Fumigation, washing, atomization and warm-compress approaches are theoretically plausible because ocular allergy develops at the conjunctival and tear-film interface. The retrieved clinical evidence was limited and under-standardized. These strategies should be discussed as translational candidates requiring controlled evaluation, not as established therapy. Essential reporting elements include formulation composition, preparation methods, temperature, exposure time, pH and osmolality, hygiene control, particulate burden, contraindications and corneal staining.

### 5.5 Acupuncture-related approaches

Acupuncture has been investigated more extensively in allergic rhinitis and asthma than in ocular allergic diseases (42). Direct ocular-allergy-specific clinical evidence remains sparse. Acupuncture is therefore more appropriately considered an indirect neuroimmune or systemic-regulatory strategy than a directly supported treatment for ocular allergy.

## 6 Comparative findings and outcome limitations

Available clinical evidence, including one secondary meta-analysis and several primary clinical studies, generally suggests higher total effective rates, greater improvement in itching and conjunctival hyperemia, or lower recurrence with TCM-related or integrated interventions (27, 34–38, 47, 48). These findings are clinically suggestive but require cautious interpretation because study designs, diagnostic criteria, formulas, comparators, follow-up, unit of analysis and adverse-event ascertainment are heterogeneous. Secondary meta-analytic evidence and the primary studies summarized in Table 3 may overlap and therefore should not be regarded as fully independent sources of evidence.

Overall, the available clinical evidence is limited by generally small or single-center samples, incomplete reporting of randomization and masking procedures, unclear allocation concealment, heterogeneous interventions, short follow-up durations, reliance on non-standard total effective rate outcomes, incomplete prospective adverse-event ascertainment, occasional eye-level analyses without clear adjustment for inter-eye correlation, and the predominance of single-country Chinese studies, all of which may increase the risk of publication bias. These limitations do not negate the clinical evidence summarized above, but they restrict causal inference, independent attribution of effect and generalizability.

The total effective rate deserves specific caution. It is not an internationally standardized ophthalmologic endpoint and may vary widely across studies. It often combines subjective symptoms, clinical signs and investigator-defined response categories into a composite outcome whose thresholds are not comparable across trials. Such composite outcomes may be described descriptively but should not be regarded as equivalent to validated ophthalmic endpoints or as sufficient evidence of clinically meaningful efficacy.

Future studies should prioritize validated ocular itching scores, conjunctival hyperemia grading, corneal fluorescein staining, tear-film parameters, recurrence after treatment discontinuation, quality-of-life measures, rescue-medication use, steroid-sparing effects, and tear-fluid or conjunctival biomarkers such as tear IgE, IL-4, IL-5, IL-13, TSLP and eosinophil-associated markers (22–25, 49–52).

## 7 Mechanistic hypotheses requiring ocular-specific validation

The immunopathology of ocular allergy provides a plausible framework for interpreting proposed mechanisms of TCM modalities. Most mechanistic evidence summarized in this review originates from non-ocular allergic disease models, general inflammatory models, pharmacological studies, *in vitro* cell systems or network-pharmacology analyses. Such evidence should be interpreted as exploratory mechanistic support rather than direct evidence for ocular allergy.

In the early allergic response, allergens cross-link IgE bound to Fc-epsilonRI on conjunctival mast cells, causing degranulation and release of histamine, tryptase, prostaglandins, leukotrienes and cytokines. These mediators drive itching, vasodilation, tearing, chemosis and inflammatory-cell recruitment (12, 14–18, 53, 54). Some herbal extracts and compounds, including Houttuynia-related preparations, have been reported in experimental systems to affect mast-cell mediator release or NF-κB-related cytokine production (30–33). These findings support plausibility but do not replace ocular clinical validation.

Type 2 inflammation is central to chronic ocular allergy. IL-4 and IL-13 promote IgE class switching and amplification of type 2 inflammation, while IL-5 supports eosinophil maturation, recruitment and survival. In VKC and AKC, eosinophilic inflammation and tissue remodeling contribute to corneal complications and persistent symptoms (5–13, 49–52, 55, 56). Proposed regulation of Th1/Th2, Th17/Treg or eosinophilic pathways by TCM modalities should therefore be tested using tear cytokines, conjunctival cytology and ocular surface imaging rather than inferred from respiratory or skin-allergy models.

The ocular surface epithelium is an active immune-regulatory structure. Allergens, pollutants, mechanical injury and preservatives can induce epithelial alarmins such as TSLP, IL-33 and IL-25, thereby promoting dendritic-cell, innate lymphoid cell, Th2-cell, mast-cell and eosinophil activation (55, 57).

**Figure 2:**
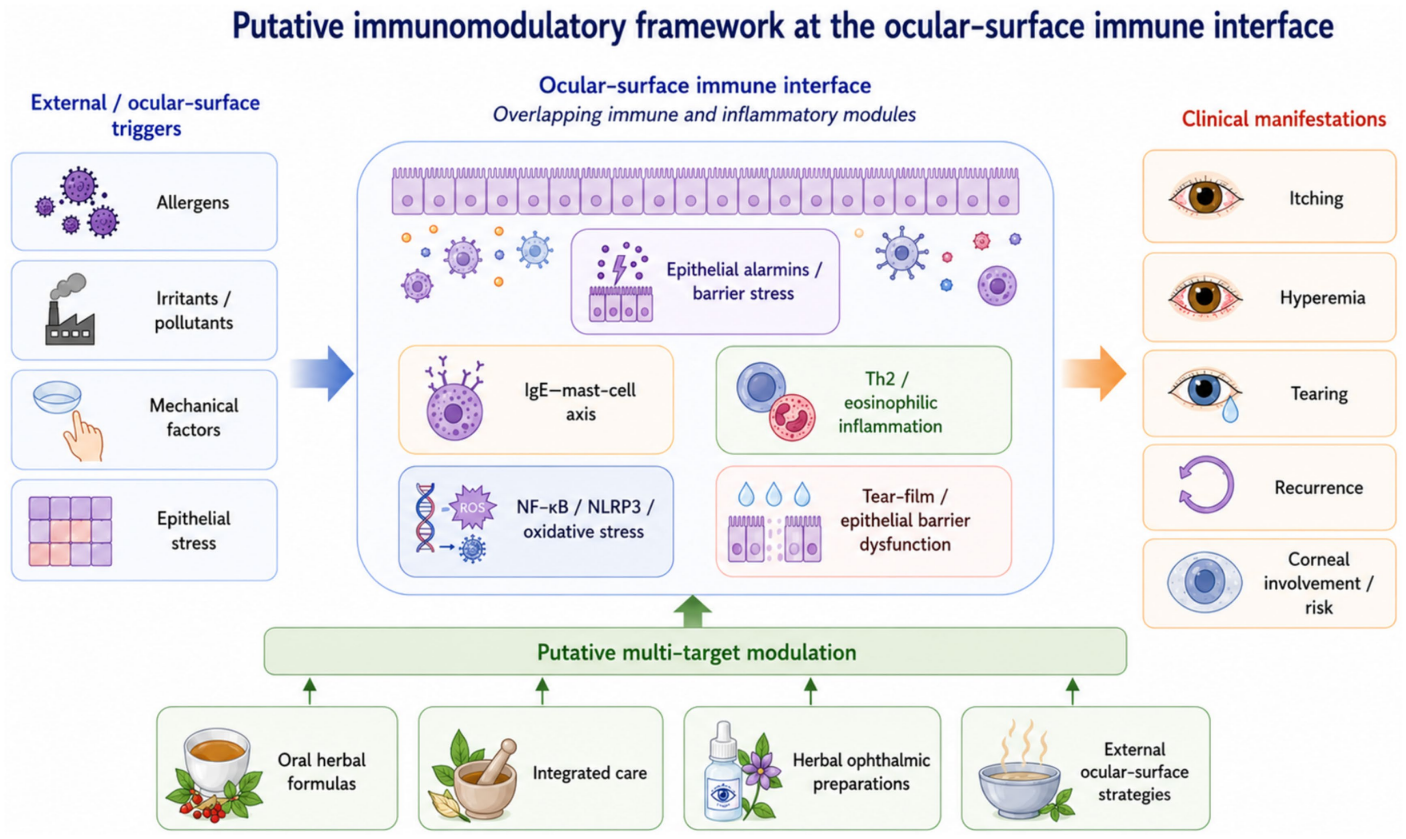
Putative immunomodulatory framework at the ocular surface immune interface. External and ocular surface triggers, including allergens, irritants or pollutants, mechanical factors and epithelial stress, may converge on overlapping modules involving epithelial alarmins, IgE-mast-cell responses, Th2/eosinophilic inflammation, NF-κB/NLRP3-related innate immune amplification, oxidative stress and tear-film or epithelial barrier dysfunction. TCM modalities are positioned as putative multi-target modulators; arrows indicate conceptual relationships and should not be interpreted as evidence of clinical efficacy.

NF-κB links epithelial stress to inflammatory transcription, whereas NLRP3 inflammasome signaling may connect oxidative stress and innate immune amplification with IL-1beta/IL-18 maturation (58–63). These pathways are relevant targets for future eye-focused testing, but current TCM evidence remains largely indirect. Figure 2 summarizes this conceptual framework, and Table 6 lists the pathway-specific assumptions and eye-focused validation endpoints.

**Table 6:** Mechanistic pathways and required eye-focused validation endpoints.

Pathway	Role in ocular allergy	TCM-related hypothesis	Current evidence status	Priority ocular endpoints
IgE-mast-cell axis	Early itching, redness, tearing and mediator release	Reduced degranulation or mediator production	Strong ocular-allergy biology; limited ocular TCM validation	Itching score, hyperemia, tear histamine/tryptase
Th2 cytokines	IL-4, IL-5 and IL-13 drive IgE and eosinophilic inflammation	Immune-balance and cytokine regulation	Mostly indirect TCM evidence	Tear IL-4, IL-5, IL-13; eosinophil markers
Eosinophilic inflammation	Important in VKC/AKC corneoconjunctival injury	Reduced recruitment and survival signals	Disease relevance strong; TCM validation limited	Conjunctival cytology, tear eosinophil proteins, corneal signs
Epithelial alarmins	TSLP, IL-33 and IL-25 initiate type 2 immunity	Reduced epithelial stress and barrier injury	Emerging and mostly indirect	Tear/conjunctival TSLP, IL-33, barrier markers
NF-κB/NLRP3/oxidative stress	Inflammatory transcription and innate immune amplification	Anti-inflammatory, antioxidant or inflammasome modulation	Plausible but often non-ocular	IL-1beta, IL-18, oxidative markers, corneal staining
Barrier dysfunction	Promotes allergen entry, tear-film instability and chronic inflammation	Support of epithelial integrity and mucin/tear-film stability	Understudied	Fluorescein staining, tear breakup time, mucin markers

## 8 Ocular surface delivery and translational safety

Safety should be considered separately for oral herbal medicine, topical herbal ophthalmic preparations and external ocular surface procedures. A central revision in this manuscript is that absence of reported adverse events in small, short-term or passively monitored studies is not interpreted as evidence of safety.

For oral herbal formulas, safety concerns include botanical identification, substitution or adulteration, contamination, hepatic and renal toxicity, cardiovascular effects, pregnancy risks, pediatric dosing, and herb-drug interactions. These risks are especially relevant when herbal formulas are combined with antihistamines, corticosteroids, calcineurin inhibitors or systemic medications.

For local ocular preparations, the threshold is higher. The conjunctiva and cornea are sensitive to microbial contamination, endotoxins, nonphysiological pH or osmolality, particles, solvents, preservatives and botanical irritants. Herbal eye drops should be evaluated as ophthalmic preparations, not as informal herbal extracts.

Minimum translational requirements include sterility testing, endotoxin control, pH, osmolality, particle control, preservative or preservative-free strategy, botanical authentication, marker-compound profiling, batch-to-batch consistency, formulation stability, packaging suitability and corneal epithelial toxicity testing (64–72).

For fumigation, washing, atomization and warm-compress approaches, safety reporting should include temperature, exposure time, preparation hygiene, filtration or particle control, pH/osmolality, contraindications, infection risk, ocular irritation and corneal staining. These methods should not be treated as benign merely because they are external or traditional.

Clinical safety endpoints should include visual acuity, slit-lamp examination, conjunctival hyperemia, corneal fluorescein staining, tear-film parameters, ocular discomfort, contact allergy, infection, intraocular pressure when corticosteroids are co-used, and follow-up after discontinuation.

In VKC or AKC with corneal risk, no herbal or integrative adjunct should delay evidence-based anti-inflammatory treatment. The corresponding safety domains and required evaluations are summarized in Table 7.

**Table 7:** Safety and translational requirements for TCM modalities.

Safety domain	Relevant route	Potential risk	Required evaluation
Systemic safety	Oral herbal formulas	Hepatic/renal toxicity, pregnancy risk, cardiovascular effects, interactions	Adverse-event recording; concomitant-drug assessment; laboratory testing when clinically indicated
Botanical identity and quality	All herbal products	Substitution, adulteration, contamination, variable marker compounds	Botanical authentication; marker-compound profiling; batch documentation
Sterility and endotoxin	Eye drops, washing/fumigation liquids	Infectious conjunctivitis or keratitis; inflammatory reaction	Sterility testing; endotoxin testing; preparation under pharmacy or good manufacturing practice conditions
pH, osmolality and particles	Topical ocular preparations	Irritation, epithelial injury, foreign-body sensation	Ophthalmic formulation testing; particle control; tolerability testing
Preservatives/excipients	Eye drops	Goblet-cell loss, epithelial toxicity, barrier disruption	Low-toxicity or preservative-free systems; ocular surface monitoring
Thermal/procedural exposure	Fumigation, washing, warm compress	Thermal injury, irritation, contamination	Standardized temperature, duration, hygiene and contraindication screening
Corneal safety	All local ocular routes	Corneal staining, epithelial defect, delayed healing	Slit-lamp examination, fluorescein staining and discontinuation follow-up
Allergic reactions	All routes	Plant allergy or contact allergy	Allergy-history screening; local and systemic adverse-event monitoring

## 9 Clinical implications for ophthalmology and integrative medicine

For ophthalmologists, the practical value of the current TCM literature is best understood as adjunctive and evidence-generating rather than as an alternative first-line treatment. Patients with mild recurrent AC may ask about herbal or integrated care, but standard topical antihistamines, mast-cell stabilizers and anti-inflammatory therapy remain the foundation of evidence-based management.

For severe ocular allergic diseases with corneal involvement, particularly VKC and AKC, clinical caution is essential. Photophobia, reduced vision, corneal staining, shield ulcer, persistent mucous discharge or repeated corticosteroid requirement should prompt ophthalmic evaluation. Any herbal eye drop, fumigation, washing or atomization approach should be evaluated with explicit ocular surface safety monitoring rather than treated as an inherently benign traditional procedure.

For future clinical research, the main opportunity is to connect traditional intervention routes with measurable ocular surface biology. Trials should report subtype-specific diagnosis, standardized botanical composition, validated symptom and sign scores, tear biomarkers, recurrence after discontinuation, conventional co-treatment and prospective adverse events.

### 9.1 Limitations and future research priorities

This review has several limitations. It is a structured narrative review rather than a systematic review or meta-analysis and does not provide pooled effect estimates or formal certainty-of-evidence grading. Although database-specific search strategies are now reported, the evidence map remains descriptive and based on publicly retrievable records. The clinical studies are heterogeneous in diagnosis, severity, formula composition, treatment duration, comparator, outcome definition and safety reporting. Many reports rely on total effective rate, which is difficult to compare with validated ophthalmologic endpoints. Mechanistic evidence often comes from non-ocular allergic diseases or general inflammatory models and should be interpreted as exploratory. Because most clinical studies originated from a single country and were published in Chinese-language journals, publication bias and selective reporting cannot be excluded.

These limitations also identify priorities for future research. Future studies should separate SAC, PAC, VKC, AKC and GPC; specify botanical composition and quality control; use validated ocular endpoints; include tear or conjunctival biomarkers; evaluate recurrence after discontinuation; and monitor ocular and systemic safety prospectively. Table 8 summarizes these priorities.

**Table 8:** Future research priorities.

Priority	Current gap	Recommended design or endpoint
Disease-subtype stratification	Evidence is concentrated in AC/SAC/PAC and VKC; AKC and GPC lack eligible direct TCM clinical studies within this review	Report SAC, PAC, VKC, AKC and GPC separately; avoid over-inference
Validated ocular endpoints	Many studies rely on total effective rate	Use itching scores, hyperemia grading, corneal staining, tear-film parameters and quality of life
Ocular biomarkers	Tear and conjunctival immune markers are rarely reported	Tear IgE, IL-4, IL-5, IL-13, TSLP, eosinophil markers and conjunctival cytology
Houttuynia eye-drop trials	Largest signals remain short-term and not confirmatory	Prospective randomized trials with formulation quality control and corneal safety monitoring
External ocular surface procedures	Fumigation/washing/atomization are under-standardized	Report composition, temperature, exposure time, pH/osmolality, particles and corneal staining
Long-term follow-up	Recurrence and seasonal relapse are rarely available	Assess recurrence after discontinuation, rescue medication and steroid-sparing effects
Mechanistic validation	Network pharmacology and non-ocular mechanisms are often over-interpreted	Test NF-κB, NLRP3, mast-cell, epithelial-barrier and tear cytokine endpoints in ocular studies

## 10 Conclusion

The evidence for TCM modalities in ocular allergy is uneven and should be interpreted with strict evidence boundaries. Direct clinical evidence identified in this review is mainly concentrated in AC/SAC/PAC and VKC. Oral herbal formulas and integrated TCM-standard therapy represent the main clinical evidence base identified within the retrieved literature, while Houttuynia eye drops provide a smaller but more directly local topical signal. No eligible direct TCM clinical evidence was identified for AKC or GPC within the search scope, and these diseases should not be included in efficacy generalizations.

Mechanistically, TCM modalities may interact with the IgE-mast-cell axis, Th2/eosinophilic inflammation, epithelial alarmins, NF-κB/NLRP3 signaling, oxidative stress and ocular surface barrier dysfunction. These mechanisms require ocular-specific validation and should not be used as substitutes for clinical evidence.

TCM-related interventions should not replace or delay established ophthalmic therapy, particularly in severe ocular allergic disease with corneal involvement or visual risk, including VKC and AKC. Future high-quality, adequately powered clinical trials with standardized formulations, validated ophthalmic endpoints and prospective safety monitoring are required before TCM modalities can be recommended more broadly in evidence-based ophthalmic practice.
